# Versajet-Assisted Hydraulic Epilation Through Small Incisions for Axillary Osmidrosis

**DOI:** 10.1007/s00266-018-1097-y

**Published:** 2018-02-20

**Authors:** Jin Ho Han, June-Kyu Kim, Kun Chul Yoon, Hyun Woo Shin

**Affiliations:** Department of Plastic and Reconstructive Surgery, Kangbuk Samsung Hospital, Sungkyunkwan University School of Medicine, 29 Saemunan-ro, Jongno-gu, Seoul, 03181 Republic of Korea

**Keywords:** Axillary osmidrosis, Apocrine glands, Hyperhidrosis

## Abstract

**Background:**

Osmidrosis is a malodorous disease caused by the breakdown of sweat secreted from the apocrine glands by surface bacteria. The aim of this study was to evaluate the effect of Versajet-assisted hydraulic epilation for the treatment of axillary osmidrosis.

**Methods:**

Thirty-two patients with axillary osmidrosis (64 axillae) underwent Versajet-assisted hydraulic epilation between January 2016 and January 2017. Subjective assessments were evaluated by a patient survey at least 3 months after the procedure.

**Results:**

There were no complications other than one mild pigmentation in the axilla at 3 months after the procedure. Thirty-two patients evaluated malodor elimination as good. No patients evaluated it as fair or poor. There were no recurrences.

**Conclusion:**

Versajet-assisted hydraulic epilation is an ideal surgical procedure for the treatment of axillary osmidrosis that decreases complications and recurrence.

**No Level Assigned:**

This journal requires that authors assign a level of evidence to each article. For a full description of these Evidence-Based Medicine ratings, please refer to the Table of Contents or the online Instructions to Authors www.springer.com/00266.

## Introduction

Osmidrosis is a malodorous disease caused by the breakdown of sweat secreted from the apocrine glands by surface bacteria [1].

There are currently no recognized diagnostic criteria for osmidrosis (bromhidrosis).

We have considered surgery when there is a subjective symptom of odor, and there is a sense of discomfort in social activities.

The various treatment methods include non-surgical ones, such as the use of topical antiperspirants or injection of botulinum toxin, and surgical ones such as surgical epilation and liposuction. The surgical methods are more effective and have long-lasting results [2].

The aim of our surgery is to remove almost every apocrine gland and reduce recurrence rates, while also ensuring minimal postsurgical complications.

If the dermis is excessively damaged to reduce the recurrence of osmidrosis, complications such as skin necrosis and hematoma may occur [1]. Thus, we used a Versajet (Versajet hydrosurgery system, Smith & Nephew, Memphis, TN, USA) to minimize dermis injury and remove almost all apocrine glands below the dermis [3]. ‘Hydraulic epilation’ means epilation with a high-pressure water jet.

The surgical results were evaluated by clinical outcomes, histopathologic findings, subjective evaluation of degree of improvement, and evaluation of surgical satisfaction.

The aim of this study is to review the literature on various surgical methods for the treatment of osmidrosis and to compare the incidence of complications and recurrence rate with the results of this study to show the effectiveness of Versajet-assisted hydraulic epilation.

## Patients and Methods

### Patients

From January 2016 to January 2017, 32 axillary osmidrosis patients underwent Versajet-assisted hydraulic epilation through small incisions.

All patients who had subjective symptoms of odor and discomfort in social life due to odor were included in the study, and those who refused the use of Versajet or did not agree with this study were excluded from the study.

The mean age of the patients was 30.4 ± 19.7 years, ranging from 11 to 71 years. The male-to-female ratio of patients who underwent the procedure was 1:5.4. The mean follow-up period was 8.16 ± 3.2 months with a minimum of 3 months of follow-up to check for recurrence.

The author used Versajet (Versajet^®^ hydrosurgery system, Smith & Nephew, Memphis, TN, USA) to remove the apocrine glands below the dermis with minimal injury. Postsurgical evaluation included histopathological evaluation, degree of improvement in symptoms and patient satisfaction.

### Surgical Procedure

**Fig. 1:**
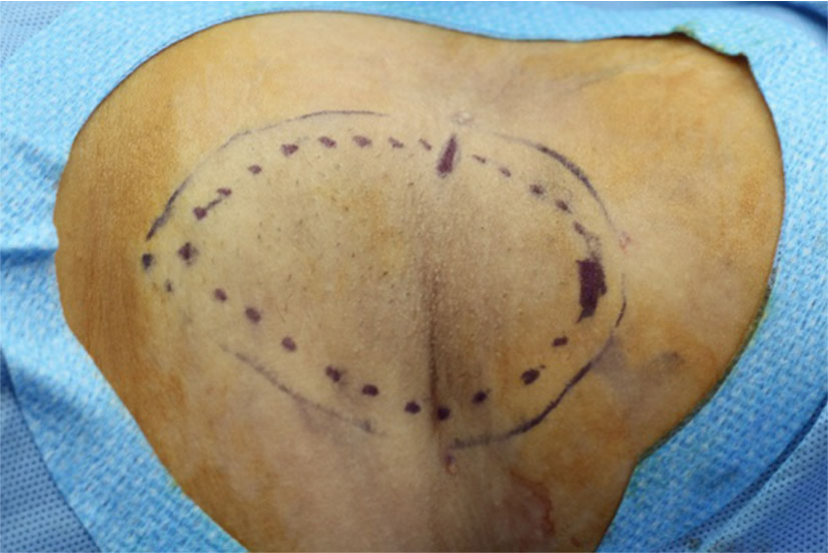
Preoperative design of axillae. Areas to be treated were marked 1 cm wider than the hair-bearing area. Two small incisions were made on the axillary crease and the anterior axillary line

Both axillae were shaved before surgery, and the subdermal undermining area was marked at a width of about 1 cm larger than the hair-bearing area of the axilla (Fig. 1). After local anesthesia using 1:100,000 lidocaine and epinephrine solution into the incision site, about 80–100 cc tumescent solution (Hartmann solution 500 cc + normal saline 25 cc + 2% lidocaine 25 cc + 8.4% bicarbonate 5 cc + epinephrine 1 cc) was injected into the subcutaneous tissues. Two 1-cm-long incisions were made along the anterior and lateral margin of the marked area, parallel to the axillary crease (Fig. 1).

**Fig. 2:**
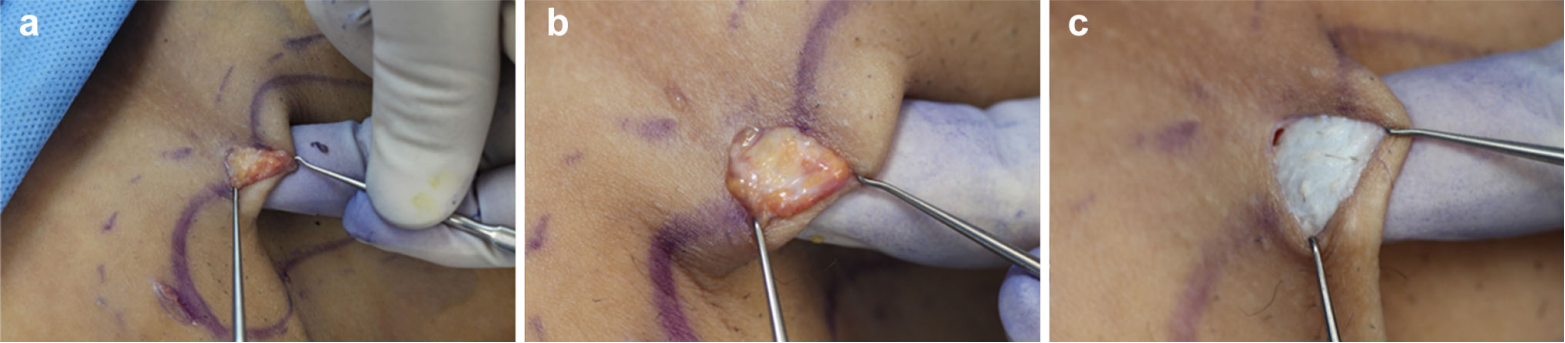
Intraoperative photographs. Subcutaneous fat and dermis were evaluated through the small incisions with 2 skin hooks. As each surgical procedure progresses, removal of fat and glands was noted. After hydraulic epilation with Versajet, the uniform dermis is observed. **a** Preoperative status, **b** after liposuction status, **c** after hydraulic epilation with Versajet status

**Fig. 3:**
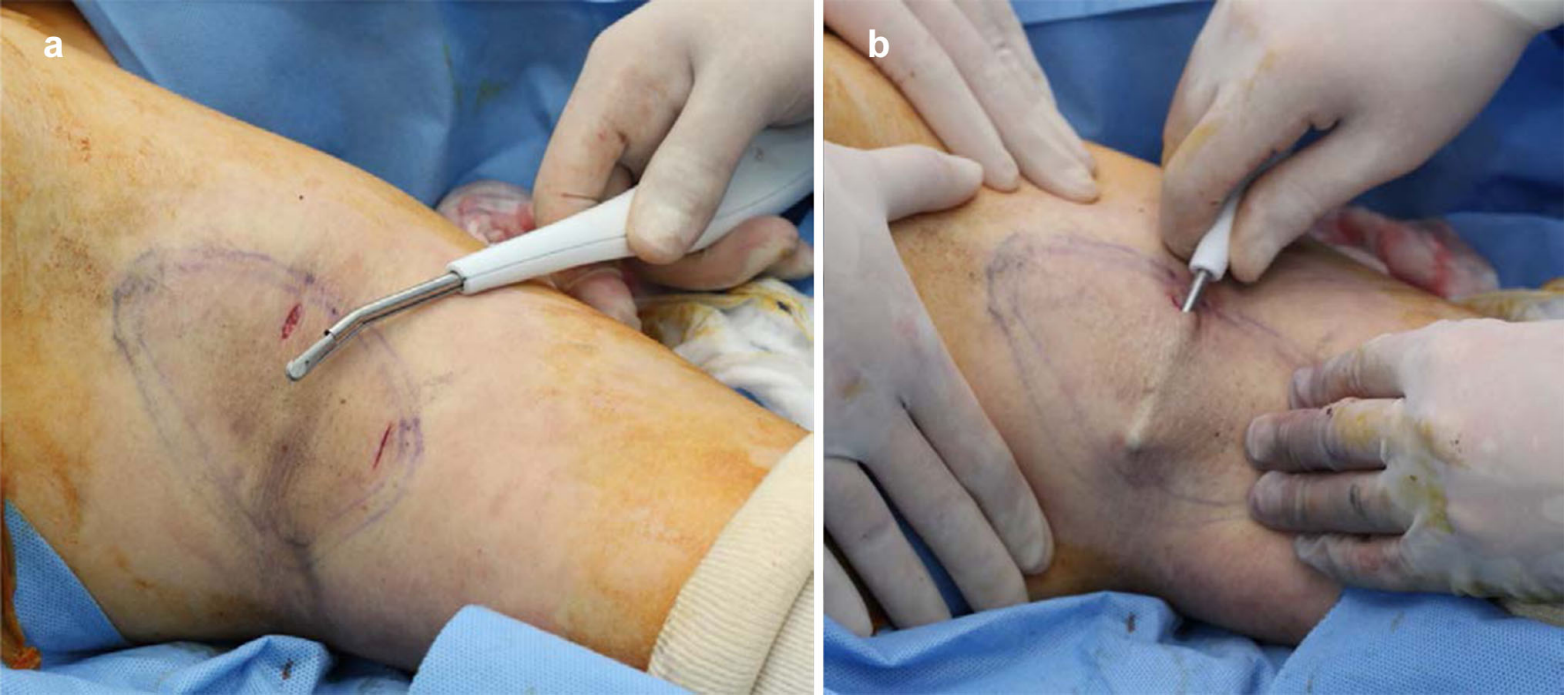
Intraoperative photographs. Versajet hand piece was turned upside down, tenting the flap and shaved the deep dermis in a crisscross manner

The axillary skin flap was undermined using scissors. And then, liposuction was used to remove fat that was unevenly attached to the dermis (Fig. 2a, b). After ensuring the uniform removal of fat, we turned the Versajet hand piece upside down, tenting the flap, set it to power grade 3, and shaved the deep dermis in a crisscross manner (Fig. 3). We set the end point of hydraulic epilation to be when all the apocrine glands and fat below the dermis, especially around the hair follicles, were removed and a uniform dermis was observed (Fig. 2c).

**Fig. 4:**
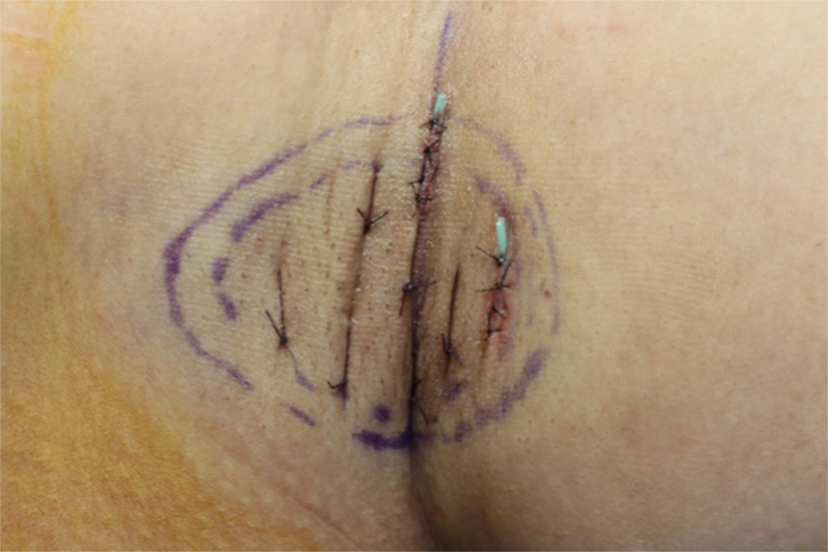
Intraoperative photograph (after quilting suture and Penrose drain insertion)

Electrocautery was used for hemostasis of axillar perforators and veins. Irrigation was performed through the incision site to remove residual gland debris and hematomas. Quilting suture was performed on the skin flap using 4-0 vicryl. And then a 3-mm-width Penrose drain was inserted into both axillae (Fig. 4). The wound was closed with 5-0 nylon and dressed with bolster and elastic bandage.

### Postoperative Management

All patients were monitored at 1, 3, 5, 10, and 14 days after the procedure. The Penrose drain was removed 1 day after the procedure. The quilting suture and compression dressing were removed 5 days after the procedure. Patients were instructed to limit the movements of their armpits and avoid rubbing and vigorous exercise. The suture at the incision site was removed approximately 10 days after the procedure.

### Histological Assessment

In 8 of the 32 patients who agreed to histopathological evaluation, tissue biopsies were obtained after each procedure (after undermining skin flap, liposuction, and hydraulic epilation).

After staining with hematoxylin and eosin, a pathologist counted the apocrine glands in the same area under ×40 and ×100 magnifications with an optical microscope.

All biopsies were performed after obtaining written informed consent from patients.

We received approval from the Institutional Review Board at Kangbuk Samsung Hospital.

### Subjective Assessments

Patient satisfaction was evaluated based on responses to a questionnaire that was administered to the patients 3 months after the surgery.

The degree of improvement in body odor was evaluated on a scale of good, fair, and poor (Tables 1, 2).

**Table 1:** Demographics of patients

Variables	Value
Number of patients	32
Age	30.4 ± 19.7 (11–71)
Sex	
Male	5 (16%)
Female	27 (84%)
Body mass index	24.46 ± 3.9 (17.4–29.5)
Family history	25 (78%)
Combined hyperhidrosis	29 (90%)
Follow-up time (month)	8.16 ± 3.2

**Table 2:** Postoperative evaluation of complications

Complications	No. of patients
Hematoma	–
Seroma	–
Wound dehiscence	–
Skin necrosis	–
Pigmentation	1
Scar contracture	–
Shoulder movement limitation	–
Total	1

The result was considered good when the patient, physician, or others around the patient were not aware of the malodor even when the patient was sweating. A fair result was a significant reduction in the malodor, but with occasional detection of the malodor by the patient when sweating. The result was said to be poor when the patient and those around were aware of the malodor. Additionally, the degree of improvement in axillary hyperhidrosis, degree of disappearance of axillary hair, and degree of postoperative satisfaction were also evaluated on a similar scale (Table 3).

**Table 3:** Postoperative evaluation

Variables	Value
Malodor elimination, axilla	
Good	32
Fair	–
Poor	–
Sweating elimination, patients	
Significant	30
Improved, not very significant	2
No change	–
Reduced hair growth	
Much (> 75%)	2
Moderate (50–75%)	28
Little (< 50%)	2
Subjective assessment	
Very satisfactory and recommend operation	29
Satisfactory	3
Regretful	–

### Statistical Analysis

Statistical analysis was performed using SPSS version 24 (IBM SPSS Inc., Chicago, IL, USA). Paired t-tests were used to analyze the changes in the number of apocrine glands in patients suffering from axillary osmidrosis, before and after liposuction and hydraulic epilation with Versajet.

## Results

The male-to-female ratio of patients who underwent the procedure was 5:27.

There were no complications other than mild pigmentation in the axilla.

Average apocrine gland counts under ×40 magnification were 66.4 preoperatively, 9.3 after liposuction, and 2.4 after Versajet-assisted hydraulic epilation (Table 4).

**Table 4:** Apocrine gland counts on axillary tissue biopsies at 40× magnification

	Preoperative apocrine gland counts	After liposuction apocrine gland counts	After Versajet apocrine gland counts
Patient 1	100	9	0
Patient 2	15	4	1
Patient 3	63	20	12
Patient 4	45	3	0
Patient 5	43	7	2
Patient 6	140	4	0
Patient 7	110	20	0
Patient 8	15	7	4
Average	66.4 (100%)	9.3 (14%)	2.4 (3.6%)

**Fig. 5:**
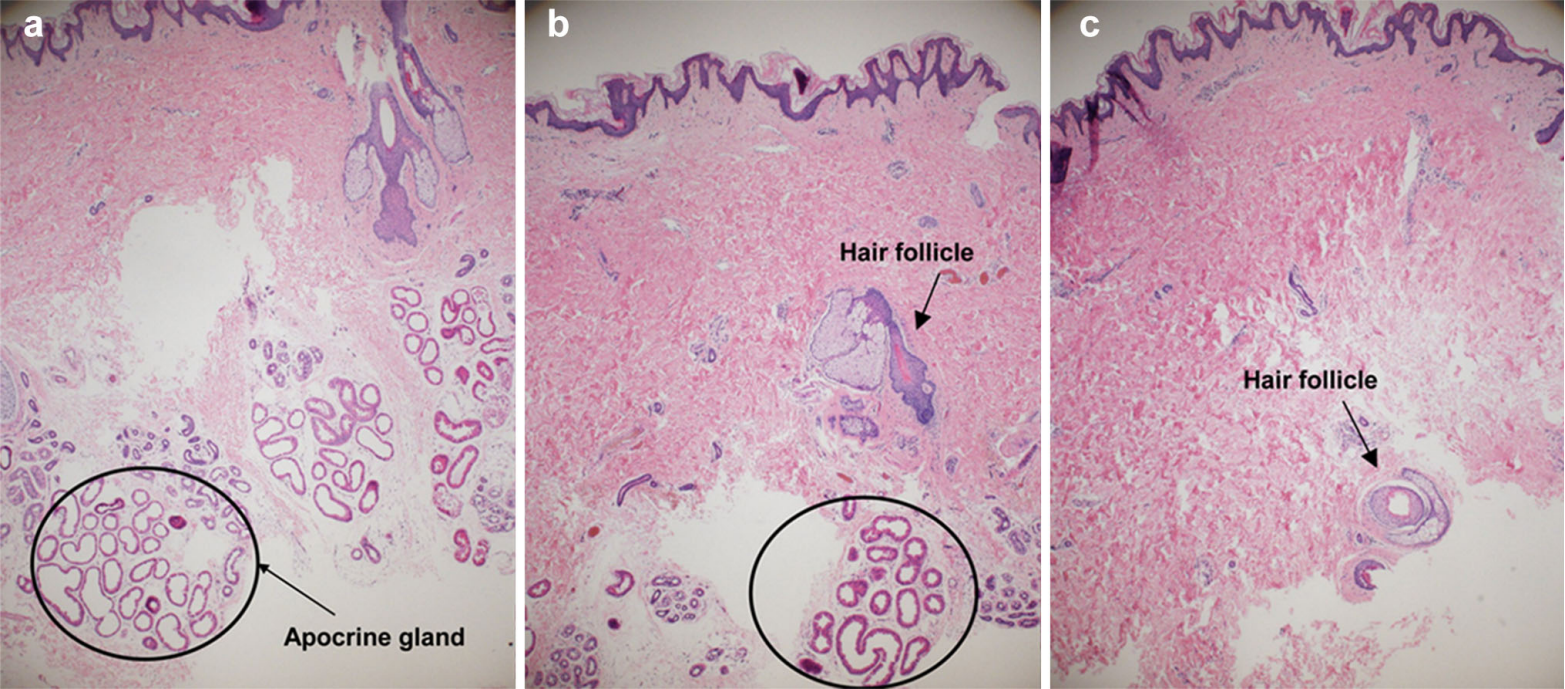
Histologic findings. Hematoxylin and eosin staining was carried out before surgery, after liposuction, and after Versajet treatment. As each surgical procedure progresses, removal of apocrine glands was noted. **a** Preoperative status, **b** after liposuction status, **c** after hydraulic epilation with Versajet status

The number of apocrine glands after liposuction was reduced significantly when compared with that before surgery. However, some glands were still found to remain in the deep dermis and around the hair follicle, but most were removed after hydraulic epilation with Versajet (Fig. 5).

Three months after the procedure, all 32 patients responded with a ‘good’ result that they were not aware of the malodor. Thirty of the 32 patients responded with a significant reduction in sweating after surgery, and 2 patients responded with a slight decrease. Two patients reported significant reduction in axillary hair growth, 28 patients reported a moderate reduction, and 2 others had a slight reduction.

All patients were satisfied with the results of the procedure. Twenty-nine patients responded that they were highly satisfied and would recommend this surgical procedure to others. Three patients, although satisfied with the procedure, did not wish to recommend it to others.

## Discussion

Various modalities have been tried for the treatment of axillary osmidrosis. Surgical epilation is known to be the most effective method for symptomatic osmidrosis. However, it is accompanied by complications such as hematoma, seroma, skin flap necrosis, wound dehiscence, and scar formation. Additionally, there is a prolonged restriction in arm movements over the course of the long recovery period. Kesselring in 1983 used liposuction as a surgical method to overcome these drawbacks. An advantage with this method is that it can be performed in an outpatient setting, with fewer wound complications, a shorter recovery time, and no dermal injury [4]. However, this method increased the incidence of recurrence due to incomplete apocrine gland removal. In previous studies, the recurrence rate was reported to be 2–57% [5], and in a study by Grazer, it was reported to be as high as 30% [6].

Tsai and Lin compared the results of simple liposuction and curettage after liposuction. Patients who underwent curettage after liposuction showed a greater degree of reduction in body odor than those who underwent only liposuction [7]. It was thought that curettage of the deep dermis using a cartilage shaver after liposuction could improve the rate of removal of apocrine glands. In case the dermal shaving is added to liposuction using a cartilage shaver, the recurrence rate and the rate of complications were 0 and 7.7%, respectively [8].

The recurrence rate and complication rates can be interpreted as the inverse proportion between the removal of the apocrine glands below the dermis and the consequent dermal injury.

Undermining of the lower part of the dermis with the Versajet maintained the dermal circulation and removed the apocrine glands directly.

In 2013, Kim et al. first used Versajet for treatment of axillary osmidrosis. They performed two long incisions, such as surgical epilation, to flip the flap and perform epilation with Versajet in the same way as surgical epilation. This treatment method considerably improved malodor in 97% of the patients, which was a distinct improvement over previously used surgical epilation methods, and the rate of complications was reported to be only 6.4% [9].

We made small incisions similar to that made for liposuction to preserve the dermal circulation and performed hydraulic epilation with Versajet.

Versajet has two apertures and is an angled hand piece shaped like a hockey stick. A high-pressure water jet is created in the aperture at its tip, which scrapes out the target tissue. The vacuum generated by the venturi effect of other aperture removes aspirates and debris to clear the operative field, thereby reducing tissue damage while efficiently removing only the desired tissue [10].

The hydraulic jet pressure can be adjusted to 10 levels based on the tissue to be removed. Smooth granulation tissue requires the lowest setting, while tougher tissues need higher settings. At the time of the osmidrosis operation, we set the power grade of Versajet to about 2–3, held the hand piece upside down, tenting the undermined flap, and removed the subdermal fat and apocrine glands in a crisscross manner.

**Fig. 6:**
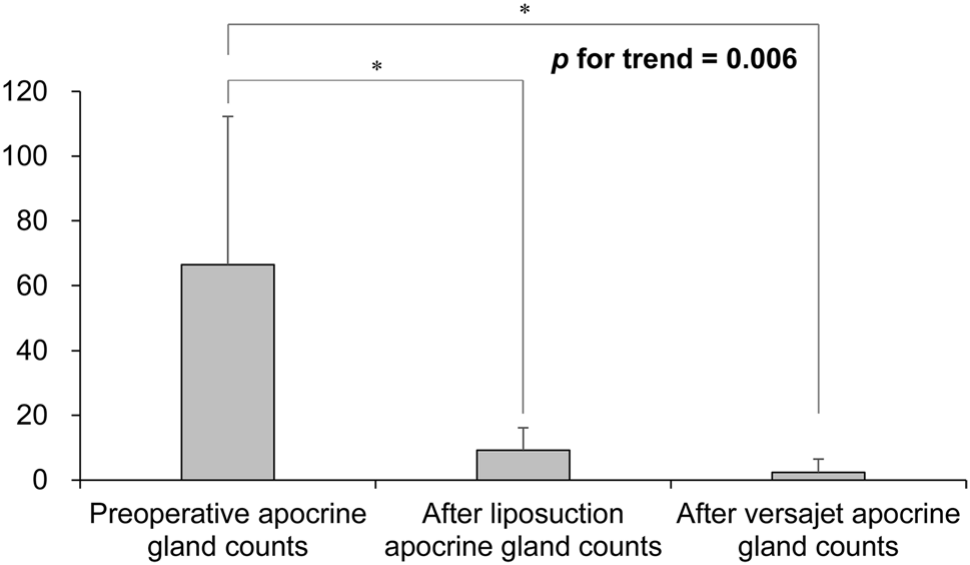
Apocrine gland counts on axillary tissue biopsy. *Statistically significant, *p* < 0.05

A quantitative analysis of apocrine glands was performed through tissue biopsies and analysis at each stage of the procedure. After liposuction, the average number of apocrine glands decreased from 66.4 to 9.3, which was an 86% reduction compared to the preoperative level. Hydraulic epilation with Versajet reduced the mean number of apocrine glands to 2.4, which was a 96.4% reduction from the preoperative level. With liposuction alone, the apocrine gland residual rate was relatively higher at 14%, which may contribute to the high recurrence rate. Liposuction when supplemented with Versajet application showed an apocrine gland residual rate of 3.5%, with no recurrence and no wound complications. The decrease in apocrine gland number at each step was statistically significant (*p* value was 0.006) (Fig. 6). Thus, hydraulic epilation using Versajet was found to be an ideal method to remove apocrine glands present under the dermis with minimum dermal injury. This can be a theoretical basis for minimizing recurrence and complications.

Two of the 32 patients relapsed after liposuction alone at a previous hospital. The remaining apocrine glands were visually confirmed by a small incision and removed directly by Versajet. No recurrence and complications reported. We believe that the use of Versajet can be useful not only in primary cases but also in recurrent cases.

Postoperative hematoma increases the incidence of skin necrosis, infection, pigmentation, and wound dehiscence and delays wound healing leading to scar formation. With Versajet, bleeding points can be directly observed over the entire surgical field through the 1-cm incision, and electrocautery can be performed. This ensures a lower likelihood of hematoma formation when compared with liposuction.

Direct visualization of the lower part of the skin flap helped to avoid overcorrection without the need for instruments like an endoscope and minimized dermal injury.

We performed quilting sutures to fix the skin flap to the wound bed to reduce the dead space and help engraft the flap to the wound bed. Particularly in thin patients with less soft tissues in the axilla, the armpit is deep and narrow, so a compression dressing using a bolster is ineffective. Therefore, it is important to induce contact between the wound bed and skin flap with a quilting suture [11].

The ratio of male to female patients who underwent the procedure was 1:5.4. These differences demonstrate that the social acceptance of body odor is affected by gender, regardless of the severity of the actual malodor. It is also expected that the gender ratio of axillary osmidrosis in patients would vary based on culture and environment (Table 5).

**Table 5:** Various treatment methods and complication of axillary osmidrosis

Author	Operative method	No. of patients	Results (%)			Surgical complications (%)	Recurrence (%)
			Good	Fair	Poor		
Inaba et al. [12]	Subcutaneous shaving	220	91.8	4.5	0.9	No mention	No mention
Wu et al. [13]	Rhomboid skin excision	102	44.1	47.1	8.8	11.1 (of patients)	2.04
Park et al. [14]	1 transverse incision and CO_2_ laser	20	80	20	0	15 (of patients)	No mention
Park et al. [15]	1 transverse incision and manual excision	48	44.8	47.9	7.3	8.6 (of patients)	No mention
Fan et al. [16]	Skin and subcutaneous tissue en bloc excision	43	95	5	–	7 (of patients)	–
Wu [8]	Suction-assisted cartilage shaver	156	92.3	5.1	2.6	7.7 (of patients)	–
Qian and Wang [17]	1 transverse incision, subdermal excision	206	97	3.4	0	50.5 (of wounds)	No mention
Rongrong Wang et al. [18]	Subcutaneous curettage	300	88	11.7	0.3	7.3 (of patients)	2
Ou et al. [5]	Superficial liposuction	20	45	50	5	15 (of wounds)	No mention
Kunachak et al. [19]	Noninvasive laser	32	81.2	12.5	6.5	0	No mention
Park et al. [20]	Very superficial ultrasound-assisted lipoplasty	21	90.5	4.7	4.7	18.8	4.7
Tsai and Lin [7]	Liposuction and curettage	10	80	20	0	–	No mention
	Simple liposuction	10	10	70	20	–	No mention
Tung [21]	Endoscopic shaver with liposuction	64	91.4	6.3	2.3	3.9 (of wounds)	2.3
Yoo et al. [22]	Endoscopy-assisted ultrasonic surgical aspiration	896	72	19	9	3.1 (of patients)	3.2
Kim et al. [9]	Versajet-assisted dermal shaving	31	97	3	0	6.4 (of patients)	No mention

A prospective study with long-term follow-ups would be ideal to evaluate the efficacy of this procedure.

## Conclusions

Versajet-assisted hydraulic epilation is an ideal surgical procedure for the treatment of axillary osmidrosis and combines the benefits of surgical epilation and liposuction. This procedure is, therefore, associated with a low recurrence rate, low incidence of complications, and minimal scarring.
